# The complete mitochondrial genome of *Pangasianodon hypophthalmus* (Sauvage 1878) (Siluriformes, Pangasiidae)

**DOI:** 10.1080/23802359.2021.1997107

**Published:** 2021-11-16

**Authors:** Wei Ni, Yihui Liu, Haigang Chen, Lingyun Yu, Wei Li, Yakun Wang, Xiaoyou Hong, Chen Chen, Ju Yuan, Fang Liu, Xiaoli Liu, Xinping Zhu

**Affiliations:** a Key Laboratory of Tropical & Subtropical Fishery Resource Application & Cultivation of Ministry of Agriculture and Rural Affairs, Pearl River Fisheries Research Institute, Chinese Academy of Fishery Sciences, Guangzhou, China; b College of Life Science and Fisheries, Shanghai Ocean University, Shanghai, China

**Keywords:** Mitochondrial genome, phylogenetic tree, *Pangasianodon hypophthalus*, Pangasiidae

## Abstract

The striped catfish, *Pangasianodon hypophthalmus* (Sauvage 1878), belongs to the family Pangasiidae, is an important economic freshwater species. We determined the complete mitogenome of the *Pangasianodon hypophthalmus* through sanger method. The complete mitochondrial genome of *Pangasianodon hypophthalmus* was a circular molecule of 16,469 bp with a total GC content of 44% which contains 37 genes, including 13 protein-coding genes, 2 rRNA genes, and 22 tRNAs genes. Phylogenetic analysis showed that *Pangasianodon hypophthalmus* clustered together with other *Pangasianodon* species and was closely related to *Pangasius larnaudii*, both species belonged to *Pangasianodon* genus.

The striped catfish, *Pangasianodon hypophthalmus* (Sauvage 1878), belongs to the order Siluriformes, family Pangasiidae, genus *Pangasianodon*, is an important economic freshwater species which is distributed in southeast Asia (Galagarza et al. 2019), and has become the main aquatic products of Vietnam at present. In addition, in several provinces of China such as Guangdong, Guangxi and Hainan, the striped catfish was also cultivated as ornamental varieties. To date, the investigations on *Pangasianodon hypophthalmus* mainly focused on immune responses (Gobi et al. 2016), growth and breeding characteristics (Legendre et al. 2000; Sattang et al. 2021), and there is no report on the complete mitochondrial genome of *Pangasianodon hypophthalmus*. Here, we reported and characterized the complete mitochondrial genome of *Pangasianodon hypophthalmus* and performed phylogenetic analysis with the mitochondrial genome of other fish species.

Fins of *Pangasianodon hypophthalmus* were collected from Baijin Aquatic Seed Co., Ltd (Sanshui, Foshan, China) (112.85E, 23.15 N). The tissue sample was kept in −80 °C refrigerator and deposited in our laboratory specimen bank (Dr. Xinping Zhu, zhuxinping_1964@163.com) under voucher number Basa_20211001 at Key Laboratory of Tropical & Subtropical Fishery Resource Application & Cultivation of Ministry of Agriculture and Rural Affairs, Pearl River Fisheries Research Institute, Chinese Academy of Fishery Sciences.

Total genomic DNA was extracted from *Pangasianodon hypophthalmus* fin tissues according to the manufacturer’s instruction of Tissue DNA Kit (Omega, USA). Then the mitogenome was sequenced through Sanger method (da Silva et al. 2020). The mitogenome of *Pangasianodon hypophthalmus* was sequenced through with 3730xl sequencer in sanger platform with seqman software at Guangzhou Tianyi Huiyuan Biotechnology Co., Ltd. Then the assembled mitochondrial genome was annotated with MitoAnnotator SeqMan from the MitoFish public database.

The complete mitochondrial genome of *Pangasianodon hypophthalmus* (GenBank accession number MZ286355.1) was a circular molecule of 16,469 bp with a total GC content of 44%, which contains 37 genes including 13 protein-coding genes (PCGs), 2 rRNA genes, and 22 tRNAs genes. The 13 PCGs included NADH dehydrogenase subunit, cytochrome c oxidase subunit, ATPase subunits and cytochrome with length ranged between 168 bp (ATP8) and 1839 bp (ND5). Most PCGs utilize ATG as their start codon, except COX1 uses GTG which was similar to most other fish species (Satoh et al. 2016). 5 PCGs including ND1, COI, ATP8, ND4L and ND5 utilize TAA as the termination codon, while other 5 PCG stop codons were incomplete, ending with T- (COII, ND4 and Cyt b) and TA- (ND2 and ND3). This feature is common among vertebrate mitochondrial protein-coding genes, and these incomplete stop codons are presumably completed as TAA (Ojala et al. 1981). Moreover, the length of 22 tRNA genes varies from 67 bp (tRNA-Ser) to 75 bp (tRNA-Leu), and the size of rRNA were 957 bp (12 sRNA) and 1681 bp (16sRNA).

The mitogenome of *Pangasianodon hypophthalmus* was aligned with that of other species using ClustalW (Thompson et al. 1994), and the phylogenetic tree was constructed using Neighbor -Joining (NJ) analysis through MEGA7 with bootstrap analysis of 1000 replicates (Kumar et al. 2016) and Bayesian Inference (BI) analysis through beast2 (Bouckaert et al. 2014). And only the Bayesian tree was given in Figure 1, as both NJ and BI tree were well supported each other and showed the same topology. *Channa argus* was used as the out-group. Phylogenetic analysis showed that *Pangasianodon hypophthalmus* clustered to *Pangasius larnaudii* and *Pangasius pangasius* and was grouped with species of genus *Pangasiidae*, which was in consistent with the previous studies in Pangasiidae (Jondeung et al. 2007; Wei et al. 2020). Moreover, the Bayesian Tree divided the Siluriformes and Perciformes into two separate groups successfully, and seven species from the Pangasiidae clustered into one branch, other species from Horabagridae, Ariinae, and Bagridae clustered into a single branch. In conclusion, the complete mtDNA sequence would establish a basis for future genetic studies of *Pangasianodon hypophthalmus* and its relative species.

**Figure 1.**
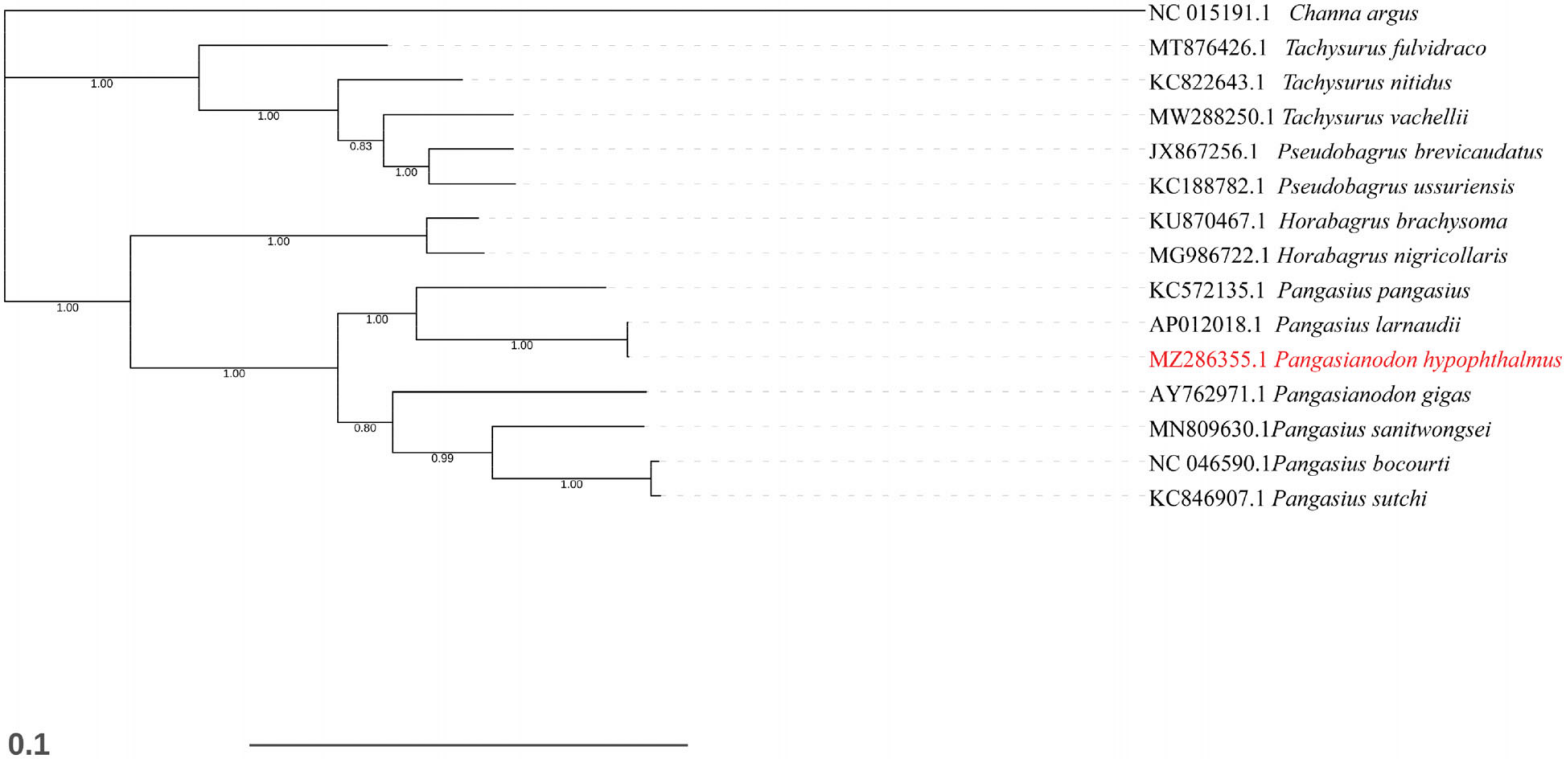
Bayesian Inference based phylogeny depicting the mitogenomic relationship between 15 fish species using *Channidae species* as outgroup. Each label includes the GenBank accession number and species name. The newly sequenced species is marked in red.

## Data Availability

The genome sequence data that support the findings of this study are openly available in GenBank of NCBI at (https://www.ncbi.nlm.nih.gov/) under the accession number MZ286355.1. The associated BioProject, SRA, and Bio-Sample numbers are PRJNA730229 (https://www.ncbi.nlm.nih.gov/bioproject/PRJNA730229), SRR14569521 (https://www.ncbi.nlm.nih.gov/sra/SRR14569521), and SAMN19225391 (https://www.ncbi.nlm.nih.gov/biosample/SAMN19225391), respectively.
